# Structures of the CDK12/CycK complex with AMP-PNP reveal a flexible C-terminal kinase extension important for ATP binding

**DOI:** 10.1038/srep17122

**Published:** 2015-11-24

**Authors:** Sarah E. Dixon-Clarke, Jonathan M. Elkins, S.-W. Grace Cheng, Gregg B. Morin, Alex N. Bullock

**Affiliations:** 1 Structural Genomics Consortium, University of Oxford, Old Road Campus, Roosevelt Drive, Oxford OX3 7DQ, UK; 2 Canada’s Michael Smith Genome Sciences Centre, British Columbia Cancer Agency, Vancouver, British Columbia V5Z 1L3, Canada; 3 Department of Medical Genetics, University of British Columbia, Vancouver, British Columbia V6H 3N1, Canada

## Abstract

Cyclin-dependent kinase 12 (CDK12) promotes transcriptional elongation by phosphorylation of the RNA polymerase II C-terminal domain (CTD). Structure-function studies show that this activity is dependent on a C-terminal kinase extension, as well as the binding of cyclin K (CycK). To better define these interactions we determined the crystal structure of the human CDK12/CycK complex with and without the kinase extension in the presence of AMP-PNP. The structures revealed novel features for a CDK, including a large β4-β5 loop insertion that contributes to the N-lobe interaction with the cyclin. We also observed two different conformations of the C-terminal kinase extension that effectively open and close the ATP pocket. Most notably, bound AMP-PNP was only observed when trapped in the closed state. Truncation of this C-terminal structure also diminished AMP-PNP binding, as well as the catalytic activity of the CDK12/CycK complex. Further kinetic measurements showed that the full length CDK12/CycK complex was significantly more active than the two crystallised constructs suggesting a critical role for additional domains. Overall, these results demonstrate the intrinsic flexibility of the C-terminal extension in CDK12 and highlight its importance for both ATP binding and kinase activity.

Cyclin-dependent kinases (CDK1-20) form a large family of serine/threonine protein kinases that depend on the binding of specific cyclin proteins for maximal catalytic activity^1^,^2^. CDKs are known for their critical roles in cell division, transcription and neuronal development, but also display more diverse functions, including control of spermatogenesis and Wnt signaling^2^,^3^,^4^. The cyclin subunit can contribute to the spatial^3^,^5^ and temporal^6^ regulation of CDK activity, as well as substrate recruitment^7^,^8^,^9^. Many CDKs additionally require phosphorylation by the CDK activating kinase (CAK; comprising CDK7/Cyclin H/MAT1 in humans), which targets a conserved threonine residue within the kinase activation loop^10^,^11^. This both stabilizes the active configuration of the CDK/cyclin complex and establishes greater interaction with the substrate^7^.

Human CDK12 (CrkRS) and its close homologue CDK13 (CDC2L5) are unusually large CDK family members (1,490 and 1,512 amino acids, respectively) containing a central kinase domain with flanking arginine-serine-rich (RS) domains^12^,^13^,^14^. Recent work indicates that these CDKs regulate transcription via assembly with the 65 kDa cyclin K1 subunit (herein CycK)^15^,^16^,^17^,^18^. The transcriptional cycle of initiation, elongation and termination is coordinated by the phosphorylation of heptad (Y_1_S_2_P_3_T_4_S_5_P_6_S_7_) repeats in the C-terminal domain (CTD) of RNA polymerase II^19^,^20^. Ser2 phosphorylation is required for transcriptional elongation and is performed by both CDK12/CycK and CDK9/CycT (P-TEFb; the positive transcription elongation factor b), which are identified as orthologues of *S. cerevisiae* Ctk1/Ctk2 and Bur1/Bur2, respectively^17^,^21^. CDK12 shows a distinct preference for CTD substrates with prior phosphorylation of Ser7 and fails to phosphorylate heptad repeats containing the common Lys7 variant^22^. CDK12 also assembles with multiple RNA processing factors^12^,^23^,^24^ and couples the transcription of pre-mRNA to 3’ end formation^25^,^26^. CDK12 complexes with cyclin L1 or L2 have also been reported to localise to nuclear speckles and to regulate RNA splicing^12^,^27^.

Most notably, CDK12 activity is critical for the expression of longer genes with a high number of exons, including key DNA damage response genes, such as *BRCA1*, *ATR*, *FANCI* and *FANCD2*^16^,^23^. Tumour suppressor function is further suggested by the presence of disabling CDK12 mutations in ovarian cancer^28^,^29^,^30^. Conversely, loss of CDK12 sensitizes cancer cells to DNA damage, making CDK12 an attractive therapeutic target for combination treatments using PARP1/2 inhibitors^29^,^31^. The first crystal structure of the CDK12/CycK complex was recently solved in in the presence of ADP providing a useful guide for structure-based drug design^22^. This work also identified an unusual C-terminal αK helix in CDK12 that was important for catalytic activity.

Here, we report further structures of CDK12/CycK complexes solved in the presence of AMP-PNP and containing variable truncations of the C-terminal kinase extension. The structure of the longest CDK12 construct showed that the C-terminal αK helix is labile and can adopt multiple conformations. Notably, its presence at the ATP-binding pocket was correlated with AMP-PNP occupancy. The structures also revealed an unusual β4-β5 loop insertion that contributed to CycK interaction. Finally, we compared the kinetic parameters of our proteins and show that the full length CDK12/CycK complex remains significantly more active suggesting additional structural elements are required for optimal function.

## Results

### Soluble expression of CDK12 requires cyclin K

**Figure 1:**
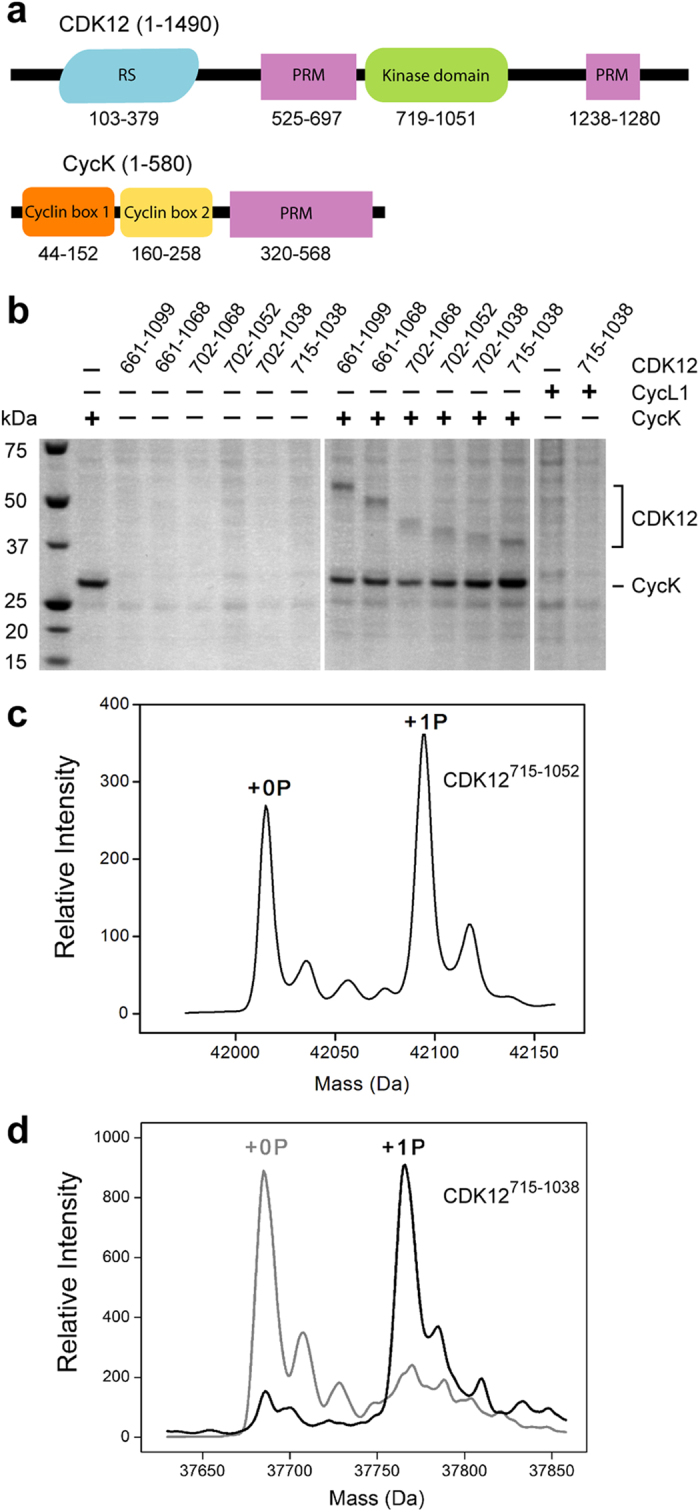
Soluble expression of CDK12 requires CycK. (**a**) Schematic showing the domain organisation of human CDK12 and CycK (RS, arginine-serine-rich; PRM, proline-rich motifs). (**b**) Small scale nickel-affinity purifications from 3 mL baculoviral expression of hexahistidine-tagged proteins. Results are shown for a subset of experiments using the indicated CDK12 constructs as well as CycK^11–267^ and CycL1^43–320^. (**c**) Deconvoluted intact mass spectra for CDK12^715–1052^ purified following co-expression with CycK^11–267^. The two mass peaks represent the native protein as well as a larger species containing a single phosphorylation on CDK12 Thr893. (**d**) Deconvoluted intact mass spectra for CDK12^715–1038^ obtained before and after treatment with recombinant CAK from *Candida albicans*. The shorter CDK12 construct is essentially unphosphorylated until CAK treatment.

Human CDK12 and cyclin K are large multidomain proteins (Fig. 1a). To prepare CDK12 protein for crystallisation trials a number of kinase domain constructs exploring truncations between residues 661 to 1099 were cloned into a modified pFastBac vector and expressed in *Sf9* insect cells. Small scale nickel-affinity purifications indicated that none of the hexahistidine-tagged constructs were solubly expressed (Fig. 1b). Cyclin-dependent kinases display inherent plasticity that may be stabilized by the binding of their cognate cyclin. Co-expression was therefore performed with suggested cyclin partners, including cyclin K1 (CycK) and cyclin L1 (CycL1). No constructs of CycL1 were identified that solubly expressed by themselves or when co-expressed with CDK12 protein (Fig. 1b). In contrast, all constructs of CDK12 were solubly expressed when co-expressed with CycK^11–267^ (cyclin domain corresponding to PDB ID: 2I53) allowing purification of the various CDK12/CycK complexes (Fig. 1b). Mass spectrometry revealed that many of the expressed CDK12 proteins were mono-phosphorylated (Fig. 1c), whereas the shorter construct CDK12^715–1038^ was essentially unphosphorylated (Fig. 1d). However, CDK12^715–1038^ was phosphorylated *in vitro* upon incubation with recombinant CAK from *Candida albicans* (Fig. 1d).

### Structure determination

For scale up, comparable expression levels of CDK12 proteins and CycK were achieved by adjusting the ratio of the two viruses used for co-expression. The expressed complexes were purified to homogeneity using nickel affinity and size-exclusion chromatography. Crystals obtained using the non-phosphorylated CDK12^715–1038^ protein exhibited multiple lattices and could not be indexed. However, diffraction quality crystals in the monoclinic space group *P*2_1_ were obtained when the same complex was phosphorylated with CAK. The resulting CDK12^715–1038^/CycK^11–267^ structure was solved by molecular replacement using CDK9 (PDB ID: 4BCG)^32^ and CycK (PDB ID: 2I53)^33^ as search models and refined at 3.15 Å resolution. Diffraction quality crystals in space group *P*2_1_ (but with different cell dimensions) were also obtained for the larger CDK12^715–1052^/CycK^11–267^ complex, which was mono-phosphorylated after expression without further CAK treatment (Fig. 1c). This structure was refined at 3.15 Å resolution and similarly contained two protein complexes in the asymmetric unit (see Table 1 for data collection and refinement statistics for both structures).

The electron density maps for both structures were generally of high quality. In the complex of the longer CDK12 construct, the CDK12 chains were traced between residues Asp718-Gln1050, with the exception of residues Asp798-Ala799 and Ser889 in chain A and residues Arg1049-Gln1050 in chain C, which were excluded from the model. CycK chains B and D were traced between residues Thr20-Met265 and Pro22-Lys262 respectively. In the more truncated structure, the electron density was most complete for the complex comprising chains A and C. CycK (chain A) was modelled between residues Pro22-Gln260 and the bound CDK12 subunit (chain C) between residues Trp719-Pro1031, with the exception of two regions of poor electron density between Gln797-Lys803 and Lys975-Lys976, respectively.

### The structures show three distinct protein complexes

**Figure 2:**
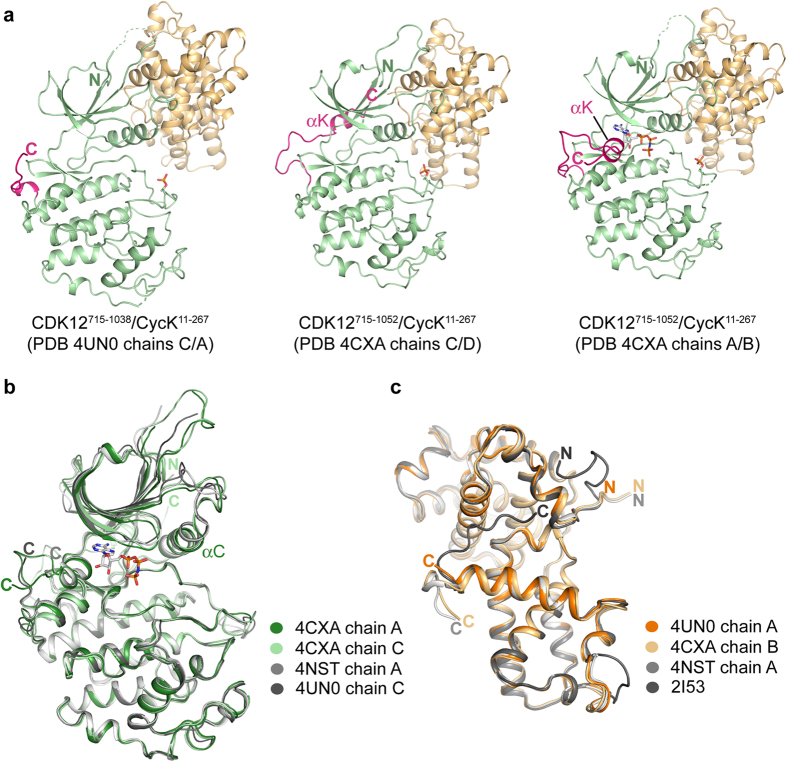
Three distinct structural states of the CDK12/CycK complex. (**a**) Ribbon representation overview of the complex structures of CDK12^715–1038^/CycK^11–267^ and CDK12^715–1052^/CycK^11–267^. CDK12 is coloured green, except for the C-terminal kinase extension shown in pink. CycK is coloured light brown. The C-terminal kinase extension, including αK, adopts distinct packing conformations in the different chains in the CDK12^715–1052^/CycK^11–267^ structure. AMP-PNP, included in all crystallisations, is observed only in chain A of this structure where its binding is stabilized by the C-terminal extension. All CDK12 subunits were phosphorylated on Thr893 (shown by sticks). (**b**) Superposition of the CDK12 chains from available crystal structures. In addition to changes in the C-terminal region, there are subtle differences in the packing of the glycine-rich loop, the β4-β5 loop and the tilt of the αC helix. (**c**) Superposition of the CycK chains from available crystal structures. The apo-structure of CycK (PDB ID: 2I53) shows differences to the complex structures in the region of the H4’ helix in the second cyclin box as well as in the N and C-termini.

The different chains in the CDK12/CycK structures share a common heterodimeric assembly of their folded domains, but exhibit significant differences in their protein C-termini yielding three distinct protein complexes (Fig. 2a). The longer CDK12^715–1052^ chains are distinguished by an additional αK helix that was also observed by Bösken *et al.*^22^ However, the current structures reveal hitherto unseen conformational plasticity in this C-terminal extension, which packs alongside the ATP pocket in chain A, but at the back of the kinase domain in chain C (Fig. 2a). Strikingly, the ligand AMP-PNP, included in all crystallisations, is bound only in chain A where its interactions are stabilized by the presence of the αK helix (Fig. 2a). Similarly, there is very poor or no electron density for AMP-PNP in the structure of the shorter CDK12^715–1038^ construct, further highlighting the importance of the αK extension.

Perhaps as a result of these varied features, other subtle differences are also observed across the available CDK12 structures. Overall, the nucleotide-bound chains show a more closed conformation of the ATP pocket due to the tighter packing of their glycine-rich loop (Fig. 2b). Closer packing of the αC helix is also observed in the CDK12^715–1052^ chains resulting in a subtle twist in their N-terminal lobe (Fig. 2b) and a small shift in the position of the bound cyclin (Fig. 2a). The structure of the CycK appears rigid and is essentially unchanged from the unbound protein^33^. Differences are observed only for the N and C-termini as well as a flexible region centred on the H4’ helix in the second cyclin box (Fig. 2c).

### Structural features of the active CDK12 kinase domain

**Figure 3:**
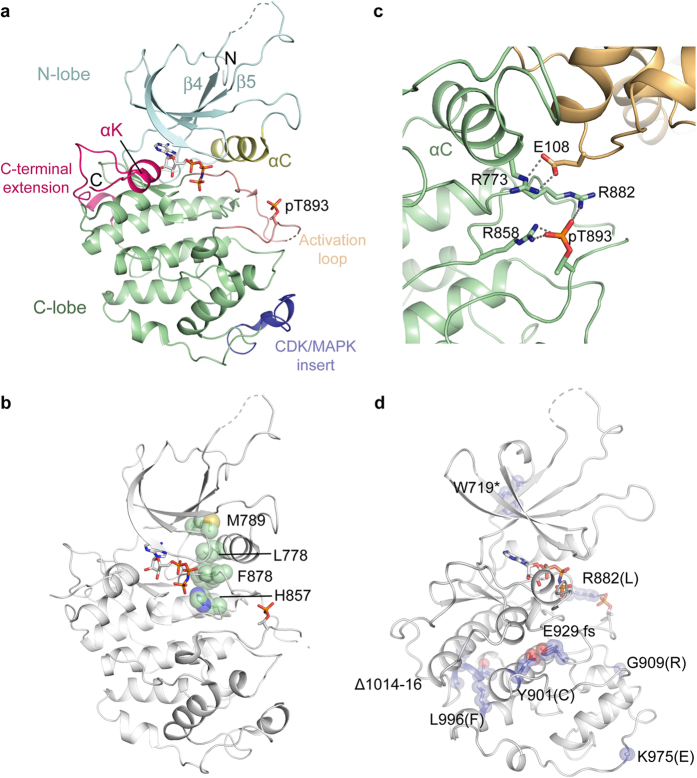
Structural features of the active CDK12 subunit (**a**) Ribbon representation highlighting selected structural features of the CDK12^715–1052^ subunit bound to AMP-PNP (PDB ID 4CXA, chain A). (**b**) CDK12 adopts an active conformation as shown by the correct alignment of the hydrophobic spine (indicated residues shown as spheres). (**c**) Phosphorylation at CDK12 Thr893 helps to stabilize the active kinase conformation. Shown are the hydrogen bond interactions of pThr893 with Arg882 (activation loop) and Arg858 (catalytic loop) as well as the nearby interaction of Arg773 (PITAIRE motif, αC helix) with CycK Glu108. (**d**) Ovarian cancer-associated mutations^28^ that impair CDK12 activity are mapped onto the structure and are predicted to destabilize the protein fold. Sites of mutation are indicated as spheres. CycK is omitted for clarity.

The CDK12 chains display an active conformation of the kinase domain consistent with their cyclin interaction and their phosphorylation at the activation loop residue Thr893 (Fig. 3a). The correct positioning of the αC helix in the AMP-PNP-bound structure is confirmed by the canonical salt bridge formed between the catalytic residues Lys756 (β3) and Glu774 (αC) (not shown). A hydrophobic regulatory spine is also established across the N and C-terminal lobes by CDK12 residues Leu778, Met789, His857 and Phe878 (Fig. 3b). Phosphorylation on Thr893 additionally stabilizes both the activation and catalytic loops through hydrogen bond interactions with Arg882 and Arg858, respectively (Fig. 3c). Arg882 is one of eight deleterious CDK12 mutations identified in ovarian cancer (Fig. 3d)^28^. Its mutation to leucine significantly impairs kinase activity^29^ and is predicted to break critical interactions between phospho-Thr893 and the activation loop.

### Interactions in the CDK12/CycK interface

**Figure 4:**
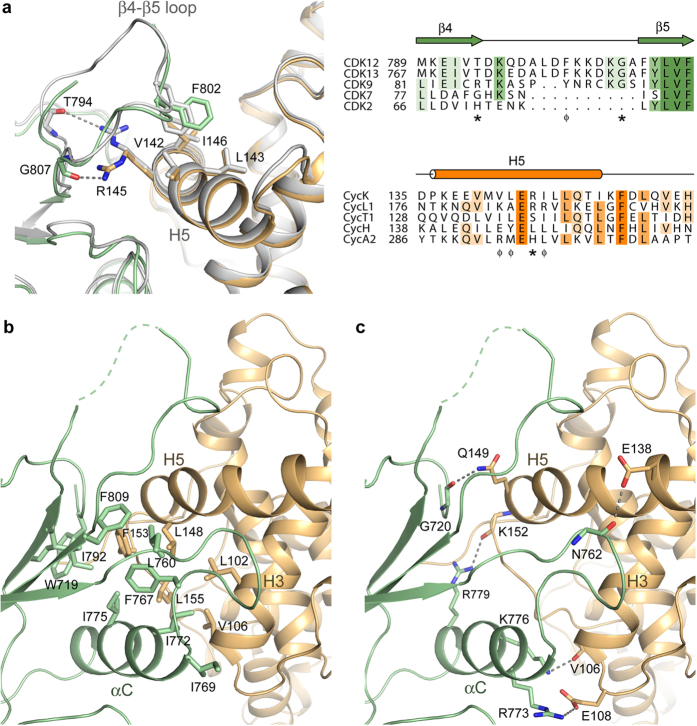
Side chain interactions in the CDK12/CycK interface (**a**) Binding of the CDK12 β4-β5 loop to CycK. Shown is a superposition of the two complexes in the asymmetric unit of the CDK12^715–1052^/CycK^11–267^ structure (CDK12 chain A and CycK chain B are coloured green and light brown, respectively, whereas chains C and D are coloured gray). A structure-based sequence alignment (right panel) reveals the insertion in the β4-β5 loop of CDK12 and CDK13 as well as the sequence divergence across this region and the interacting H5 helix of CycK. The positions of displayed residues participating in hydrophobic (ϕ) and hydrogen bond (*) interactions are marked under the alignment. (**b**) Hydrophobic interactions define the core of the protein-protein interface. (**c**) Electrostatic interactions cluster outside the core interface.

Overall, the binding of CDK12 and CycK fits the model of the transcriptional CDKs first established by the structure of the CDK9/CycT1 complex^34^. As expected for this CDK class, interactions in the protein-protein interface are limited to the kinase N-lobe and the first cyclin box motif (Fig. 2a). The new structures reveal a large β4-β5 loop, which forms a notable insertion in CDK12 and contributes additionally to the overall binding surface area (Fig. 4a). The β4-β5 loop sits atop CycK where it inserts CDK12 Phe802 into a nest of hydrophobic residues, including Val142, Val143 and Ile146 from CycK H5. This packing is further stabilized by the intervening CycK residue Arg145, which adopts two alternative rotamers in the asymmetric unit to hydrogen bond with CDK12 Thr794 or Gly807, respectively (Fig. 4a). Such heterogeneity likely reflects the intrinsic flexibility of the β4-β5 loop, which has few additional interactions except for crystal packing contacts. Below the β4-β5 loop the CDK12 ‘PITAIRE’ helix (αC) packs between CycK helices H3 and H5 (Fig. 4b). Here, the core of the protein-protein interface is hydrophobic with contributions from the kinase N-terminus (Trp719) as well as the αC and surrounding β-sheet (Fig. 4b). Beyond the hydrophobic core there are a number of electrostatic interactions that make up the periphery of the binding interface, including a salt bridge between CDK12 Arg773 (αC) and CycK Glu108 (H3) (Fig. 4c).

### Conformational plasticity of the C-terminal kinase extension

**Figure 5:**
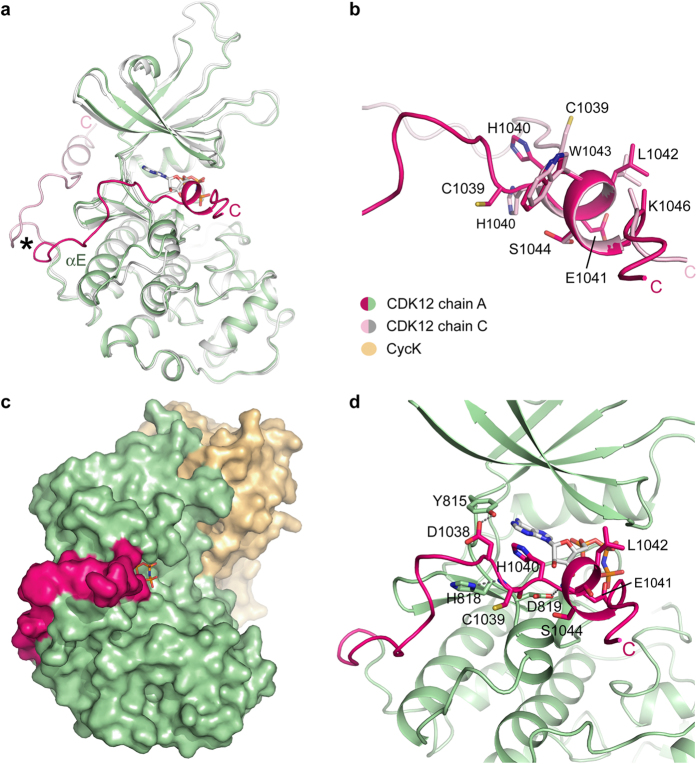
Alternative conformations of the CDK12 C-terminal extension. (**a**) Superposition of the two CDK12^715–1052^ subunits in the asymmetric unit (PDB ID: 4CXA). An asterisk marks the point at which the two chains diverge following Leu1025. In chain A (green and bright pink), the C-terminal extension folds in front of the ATP pocket, whereas in chain C (gray and pale pink) the C-terminus extends across the back of the kinase domain. (**b**) Superposition reveals that the αK helix is stably formed in both chains despite their alternative packing arrangements. (**c**) Molecular surface representation of chains A and B highlighting the enclosure of the bound AMP-PNP molecule by the C-terminal kinase extension. Coloured as in Fig. 2. (**d**) Selected interactions of the C-terminal kinase extension packing at the front of the ATP pocket (CDK12 chain A).

An unexpected feature of the CDK12^715–1052^/CycK^11–267^ complex structure is the conformational plasticity of the C-terminal kinase extension. The conformation of the two CDK12 molecules in the asymmetric unit diverges following Leu1025, which packs against the αE helix at the back of the kinase domain (Fig. 5a). The AMP-PNP-bound complex adopts a similar conformation to that reported previously by Bösken *et al.*^22^ In these structures, the C-terminal kinase extension wraps around the front of the ATP pocket where it runs parallel to the β6 strand before turning away in a perpendicular direction (Fig. 5a). By contrast, the C-terminus in the second CDK12 complex continues a path across the back of the kinase domain to pack within 4.3 Å of the bound CycK subunit (Fig. 5a). Interestingly, the αK helix, encompassing residues 1040-HELWS-1044, is formed in both CDK12 conformations suggesting that this secondary structural element is stably folded (Fig. 5b).

The C-terminal kinase extension has been shown to be critical for CDK12 activity^22^. When engaged by the ATP pocket this element acts to enclose the bound AMP-PNP molecule adding significantly to the pocket surface area (Fig. 5c). As well as contacts by His1040 and Glu1041 to the AMP-PNP moiety, there are stabilizing interactions with the kinase hinge region, including a hydrogen bond between Asp1038 and the hinge residue Tyr815 (Fig. 5d). Van de Waals interactions with the N-lobe β1 strand are additionally established by both His1040 and Leu1042. Finally, there are hydrophobic and electrostatic interactions with the C-lobe, including a hydrogen bond between the main chain amide of Glu1041 and side chain of Asp819. The current work also extends the visible structure to the polybasic tail region comprising 1045-KKRRRQ-1050, which forms a putative flexible interaction site for capture of the CTD substrate.

### Full length CDK12/CycK is required for maximal activity

**Figure 6:**
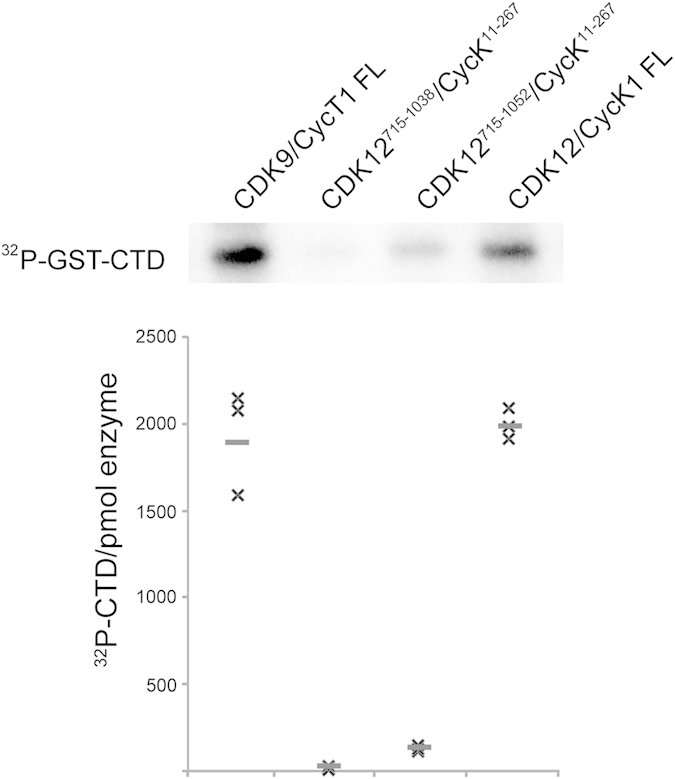
CDK12 truncations show diminished activity against the RNA Pol II CTD. Comparison of *in vitro* kinase activity against a GST-CTD substrate by various CDK12 complexes and CDK9/Cyclin T1 (FL denotes full length proteins). A representative autoradiograph detecting ^32^P-GST-CTD product is shown together with a graphic representation of 3 biological replicates of each phosphorylation reaction.

**Figure 7:**
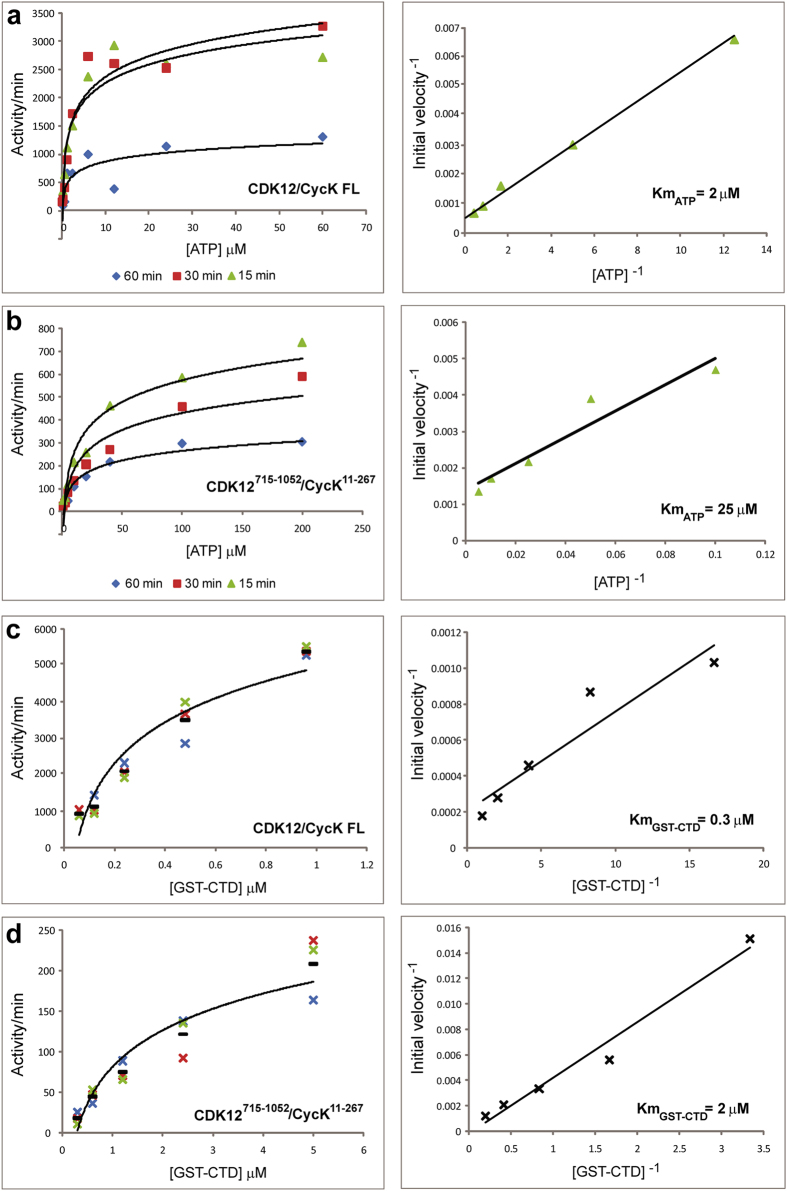
Determination of CDK12 kinetic parameters. (**a**) Full length CDK12/CycK1 activity was measured against varying concentrations of ATP over 3 time points (15 min, 30 min and 60 min). Velocity and Lineweaver-Burke plots are shown (left and right panels, respectively). Km_ATP_ was determined to be 2 μM +/− 0.2 μM ATP (S.D.) based on two independent experiments using three different preparations of the full length enzyme. (**b**) Equivalent ATP titrations and plots for the CDK12^715–1052^/CycK^11–267^ complex. Km_ATP_ was determined to be 25.5 μM +/− 0.01 μM ATP (S.D.) based on two independent experiments. (**c**) Full length CDK12/CycK1 activity was measured against varying concentrations of GST-CTD substrate using 10 μM ATP and a reaction time of 15 min. Experiments were performed in triplicate (velocity plot, left panel) and the average values plotted in a Lineweaver-Burke plot (right panel). Km_CTD_ was determined to be 0.3 μM +/− 0.06 μM (S.D.). (**d**) Equivalent GST-CTD titrations for the CDK12^715–1052^/CycK^11–267^ complex. Km_CTD_ was determined to be 2 μM +/− 0.7 μM (S.D.). Differences in the Km values between the full length and truncated CDK12 complexes suggest that other domains within the full length protein may contribute to substrate binding and turnover.

*In vitro* kinase assays using a GST-CTD substrate were performed to compare the activities of the crystallised CDK12/CycK complexes against the full length proteins. The activity of the full length CDK12/CycK1 complex was comparable to that of the equivalent CDK9/CycT1 complex (Fig. 6). By contrast, the complex comprising CDK12^715–1052^ exhibited a 10-fold reduction in activity, while the activity of the CDK12^715–1038^ truncation was severely diminished (Fig. 6). Additional kinetic analyses were performed to further understand the effects of the CDK12 truncation (Fig. 7). The determined Km values for ATP were in the typical range for protein kinases. However, the full length CDK12/CycK1 complex exhibited a Km_ATP_ value of 2 μM that was notably improved relative to the CDK12^715–1052^/CycK^11–267^ complex, which had a Km_ATP_ of 25 μM (Fig. 7a,b). Similar differences in respective binding were observed for the CTD substrate. Whereas the full length complex showed a Km_CTD_ value of 0.3 μM, the binding of the truncated CDK12^715–1052^ complex was poorer with a Km_CTD_ value of 2 μM (Fig. 7c–d). These differences suggest that other domains within the full length proteins make important contributions to the substrate interactions.

## Discussion

The structures provide three different snapshots of the CDK12/CycK complex in its phosphorylated active configuration. The most significant conformational changes occur in the C-terminal kinase extension, which features a large flexible linker followed by the unusual αK helix. Plasticity in this region is consistent with earlier observations for CDK9^35^. This CTD-directed kinase contains a C-terminal tail that folds similarly across the ATP-binding site, although it displays a distinct αK position. Flexibility in this tail has been suggested to facilitate the successive opening and closing of the ATP pocket to allow for cycles of ADP release and ATP capture^35^. Indeed, AMP-PNP was stably bound in our CDK12 structures only when trapped by the αK, while the capacity for this helix to transiently dissociate was illustrated by the folding of the other CDK12 chains. In the current crystal form, the dissociated linker and αK were folded across the back of the kinase domain in a manner reminiscent of the MAP kinases^36^. However, the limited number of specific contacts suggests that this region may be free in solution to explore multiple conformations. It would be of interest to investigate these motions in future by molecular dynamics simulations.

The CDK12 kinase acts late in the transcriptional cycle and is expected to engage a negatively charged CTD substrate^22^. The final C-terminal positions in the CDK12^715–1052^ structure form a polybasic cluster that is also loosely conserved in CDK9. The folding of the C-terminal extension across the ATP pocket is therefore also hypothesized as an adaptation to support the recruitment of the CTD substrate by provision of a complementary charged surface^22^,^35^. Dynamic movement of the C-terminus may also promote recruitment by increasing the potential search space for protein-protein interactions. Further biochemical and structural analyses are required to validate these hypotheses.

It is also striking that our truncated CDK12/CycK complexes are significantly less active than the full length proteins. Thus, there must be additional roles for other domains and likely also for other proteins in the cell. Notably, CDK12 contains multiple arginine-serine-rich (RS) motifs that may facilitate the recruitment of RNA processing factors for the coupling of transcription and RNA splicing. Proline-rich motifs (PRMs) are also identified in both CDK12 and CycK that may form recognition sites for SH3 or WW domain adaptors. It may be expected that a multiprotein CDK12/CycK complex will remain closely associated with the CTD to allow for the successive phosphorylation of multiple heptad repeats.

The CDK12/CycK structures also reveal further details of this most critical protein-protein interaction. The cyclin binding interface of the transcriptional CDKs is notably smaller than those regulating the cell cycle. We observe that this loss is offset slightly in CDK12 by an unusual β4-β5 loop insertion that packs atop the H5 helix of the first cyclin box motif. Moreover, this loop is conserved in the CDK13 kinase domain, which also binds to the CycK protein. However, a key interaction missing in the transcriptional CDK class is a contact between the cyclin and the kinase activation segment. Indeed, the cyclin interaction in these kinases is restricted to the kinase N-lobe. Structural studies of the CDK9/CycT1 complex have revealed that these interactions are instead mediated by other accessory proteins that direct kinase activity, such as HIV-1 Tat^37^. In this quaternary complex, the Tat protein contacts both the CDK9 and CycT1 subunits and modulates their protein-protein interaction. The kinase activation segment in CDK12 is similarly exposed and available to bind other partners. It will be interesting to discover if any such factors are identified that parallel the CDK9 interaction of HIV-1 Tat.

Overall, this work extends our understanding of the structural mechanisms that determine the activity of the CDK12/CycK complex. Further, it emphasizes the importance of protein flexibility as well as the contribution of regulatory elements outside the core catalytic domains. Finally, the packing of the αK helix offers a novel ATP pocket environment for the design of specific inhibitors targeting this important kinase in the DNA-damage response.

## Methods

### Cloning

For structural studies, various constructs of human CDK12 (Uniprot Q9NYV4), human CycK (Uniprot O75909) and human CycL1 (Uniprot Q9UK58) were cloned into the baculoviral transfer vector pFB-LIC-Bse by ligation-independent cloning. The vector encodes an N-terminal hexahistidine tag and a Tobacco Etch Virus Protease A (TEV) cleavage site. Bacmid DNA was prepared from *Escherichia coli* strain DH10Bac and used to generate baculovirus in Sf9 insect cells. Full length CDK12 Isoform 1 (NM_016507.2) and CycK1^15^ were cloned into pDONR221 and recombined into bacmids using the Invitrogen Baculovirus Expression System with Gateway Technology. The full length proteins were N-terminally tagged with GST or 6xHis epitopes prior to bacmid generation as per manufacturer’s conditions (Invitrogen pDEST10 and pDEST20 vectors).

### Protein expression and purification

Baculovirus for different CDK12 and cyclin constructs were used to co-infect Sf9 cells grown in suspension to a density of 2 × 10^6^ cells/mL. For small scale testing, 3 mL cultures were grown using a 24-well block and harvested 72 hours post-infection. Cells were lysed by sonication in binding buffer (50 mM HEPES pH 7.5, 500 mM NaCl, 5% glycerol, 5 mM Imidazole, 0.5 mM TCEP) supplemented with protease inhibitor cocktail set III (Calbiochem) and the lysate clarified by centrifugation. The expressed proteins were purified using nickel-sepharose resin (GE Healthcare) and analysed by SDS PAGE. Proteins for crystallisation were expressed similarly at the 1L scale. After sonication, polyethylenimine was added to a final concentration of 0.15% to precipitate DNA and the cell lysate clarified by centrifugation at 21,000 RPM for 1 hour at 4 °C. CDK12/CycK complexes were purified initially using nickel-sepharose chromatography (GE Healthcare) and eluted stepwise with imidazole before tag cleavage with TEV protease. Complexes containing CDK12^715–1038^ were additionally phosphorylated using recombinant Cak1 from *Candida albicans*. For final clean up, proteins underwent reverse nickel-affinity purification as well as size exclusion chromatography using a HiLoad Superdex S75 26/60 column (GE Healthcare) buffered in 50 mM HEPES pH 7.5, 300 mM NaCl and 1 mM TCEP.

For expression of the full length CDK12/CycK1 complex, baculoviruses for the epitope-tagged CDK12 and CycK1 were co-infected at a ratio of 4:1 and incubated at 28 °C with shaking at 150 rpm. Cells were harvested 48–72 hours post infection. The full length protein complexes were purified by tandem affinity chromatography using either combinations of 6xHis-CDK12/GST-CycK1 or GST-CDK12/6xHis-CycK1. Cell pellets were resuspended in CDK lysis buffer (10 mM Tris-HCl pH 7.5, 10 mM NaCl, 2 mM β-mercaptoethanol, 0.5 mM EDTA, 10 mM β-glycerolphosphate, 0.5 mM sodium orthovanadate, 2 mM NaF, 0.2% v/v NP-40 and EDTA-free complete protease inhibitor cocktail (Roche))^38^. The lysate was incubated on ice for 30 minutes with an additional 0.5 M NaCl and occasional manual mixing. Lysate was then subjected to sonication and clarified by centrifugation. Proteins were captured overnight at 4 °C using Ni-NTA agarose (Qiagen) that was pre-equilibrated with CDK equilibrium buffer (10 mM Tris-HCl pH 7.6, 500 mM NaCl, 10% glycerol, EDTA-free complete protease inhibitor cocktail). The Ni-NTA agarose was washed 3× with CDK equilibration buffer and the bead slurry transferred to a disposable column for step-wise elution using CDK equilibration buffer supplemented with 15, 25, 100 and 200 mM imidazole. The eluted protein complexes were dialyzed overnight against CDK activation buffer (12.5 mM Tris-HCl pH 7.5, 150 mM NaCl, 10 mM MgCl_2_, 1 mM EGTA, 5 mM β-glycerolphosphate, 0.5 mM sodium orthovanadate, 2 mM DTT, 0.01% Triton X-100, 10% glycerol, EDTA-free complete protease inhibitor cocktail) to facilitate buffer exchange and removal of imidazole. The protein was concentrated using an Amicon filtration device with a 30 KDa molecular weight cut-off and the retentate was incubated with 500 μM ATP at 30 °C for 1 hour to allow auto-activation of the CDK12 kinase. Subsequently, the proteins were further purified using glutathione agarose beads (Pierce). Following overnight batch binding at 4 °C, the beads were washed 4x with CDK activation buffer and the bead slurry transferred to a disposable column. Bound protein was eluted in a step-wise fashion with elution buffers (100 mM Tris-HCl pH 7.5, 300 mM NaCl, 1.0 mM EDTA, 0.04% Triton X-100, 4 mM DTT) supplemented with 10 mM and 20 mM glutathione. The purity of the eluted fractions was confirmed by SDS-PAGE or Western blotting before storage at −80 °C in 50 mM Tris pH 7.5, 150 mM NaCl, 0.5 mM EDTA, 0.02% Triton X-100, 2 mM DTT, 50% glycerol. Protein concentration was determined by Bradford assay.

### Kinase assays

Kinase assays were performed as described previously^15^. In brief, purified CDK complexes were incubated in kinase assay buffer (50 mM Tris pH 7.5, 100 mM NaCl, 10 mM MgCl_2_, 0.1% v/v NP-40, 1 mM DTT, 20 μM β-glycerophosphate, EDTA-free complete protease inhibitor cocktail) with varying amounts of cold ATP/ [γ-32 P]ATP and GST-CTD substrate which contains all 52 heptad repeats of human RNA Pol II C-terminal domain^15^. Reactions were incubated in a circulating 30 °C water bath for 15, 30 or 60 minutes. Kinase reactions were stopped with the addition of 6× SDS-PAGE loading dye. Samples were heated at 85 °C for 5 minutes and resolved by 4–12% SDS-PAGE. Gels were subsequently dried on 3 MM Whatmann paper and imaged with a FujiFilm FLA-7000 scanner. Phosphorylated bands were quantified using FujiFilm MultiGauge™ software.

### Mass spectrometry

Protein masses were determined using an Agilent LC/MSD TOF system with reversed-phase high-performance liquid chromatography coupled to electrospray ionization and an orthogonal time-of-flight mass analyser. Proteins were desalted prior to mass spectrometry by rapid elution off a C3 column with a gradient of 5–95% isopropanol in water with 0.1% formic acid. Spectra were analysed using the MassHunter software (Agilent).

### Crystallisation

The CDK12^715–1038^/cyclin K^11–267^ complex was buffered in 50 mM HEPES pH 7.5, 500 mM NaCl, 5% glycerol, 5 mM imidazole, 0.5 mM TCEP, 10 mM DTT and concentrated to 4.3 mg/mL. The non-hydrolyzable ATP analogue, adenylyl imidodiphosphate (AMP-PNP) was added to a final concentration of 1 mM. Crystals were grown at 4 °C in 150 nL sitting drops mixing 100 nL protein solution with 50 nL of a reservoir solution comprising 20% PEG3350, 0.1 M Bis-tris propane pH 6.5, 0.2 M sodium nitrate, 10% ethylene glycol and 1 mM MgCl_2_. Before mounting, crystals were cryo-protected with mother liquor supplemented with an additional 15% ethylene glycol and vitrified in liquid nitrogen. The CDK12^715–1052^/cyclin K^11–267^ complex was buffered in 50 mM HEPES pH 7.5, 300 mM NaCl, 1 mM TCEP, 10 mM DTT, 5 mM L-arginine, 5 mM L-glutamate and concentrated to 6 mg/mL. The ATP analogue AMP-PNP was added to a final concentration of 1 mM together with 5 mM MgCl_2_. Crystals were grown at 20 °C in 150 nL sitting drops mixing 50 nL protein solution with 100 nL of a reservoir solution comprising 20% PEG3350, 150 mM DL-malic acid. Before mounting, the crystals were cryo-protected with mother liquor supplemented with an additional 15% ethylene glycol, 3 mM AMP-PNP, 5 mM MgCl_2_ and vitrified in liquid nitrogen.

### Structure determination

Diffraction data were collected at 100 K on Diamond Light Source beamline I24 for the CDK12^715–1038^/cyclin K^11–267^ complex and beamline I02 for the CDK12^715–1052^/cyclin K^11–267^ complex. Data were indexed and integrated using XDS^39^ and scaled using AIMLESS^40^ in the CCP4 suite of programs^41^. Phases were found using molecular replacement in PHASER^42^ and search models generated by CHAINSAW^43^. The structures of CDK9 (PDB ID: 4BCG)^32^ and cyclin K (PDB ID: 2I53)^33^ were used as search models for the CDK12^715–1038^/cyclin K^11–267^ complex. The resulting CDK12 and cyclin K structures were then used as search models for the CDK12^715–1052^/cyclin K^11–267^ complex. Models were built initially using COOT^44^ and then refined and modified using alternate rounds of REFMAC5^45^ and COOT, with the later rounds of refinement in PHENIX^46^. The refined structures were validated with MolProbity^47^ and the atomic coordinate files deposited in the Protein Data Bank. Structure figures were prepared with PyMOL^48^.

## Additional Information

**Accession codes**: Atomic coordinates and structure factors have been deposited in the Protein Data Bank under accession numbers PDB ID: 4UN0 and PDB ID: 4CXA.

**How to cite this article**: Dixon-Clarke, S. E. *et al.* Structures of the CDK12/CycK complex with AMP-PNP reveal a flexible C-terminal kinase extension important for ATP binding. *Sci. Rep.* **5**, 17122; doi: 10.1038/srep17122 (2015).

## Figures and Tables

**Table 1:** Diffraction data collection and refinement statistics.

Complex	CDK12^715–1038^/cyclin K^11–267^	CDK12^715–1052^/cyclin K^11–267^
PDB accession code	4UN0	4CXA
*Data Collection*		
Beamline	Diamond Light Source, I24	Diamond Light Source, I02
Wavelength (Å)	0.9686	0.97868
Resolution^a^ (Å)	57.82–3.15 (3.37–3.15)	30.53–3.15 (3.37–3.15)
Spacegroup	*P*2_1_	*P*2_1_
Cell dimensions	*a *=* *49.6*, b *=* *148.8*, c *=* *92.2 Å	*a *=* *69.8, *b* = 138.8, *c* = 71.9 Å
	α = γ = 90.0°, β = 94.2°	α = γ = 90.0°, β = 105.0°
No. unique reflections^a^	22,936 (4145)	22,908 (4132)
Completeness^a^ (%)	99.4 (99.3)	99.8 (99.9)
I/σ(I)^a^	8.7 (2.0)	6.4 (1.5)
*R*_merge_^a^	0.19 (0.87)	0.19 (0.98)
Redundancy^a^	4.3 (4.2)	5.0 (4.9)
*Refinement*		
ligands	—	AMP-PNP
No. atoms in refinement	8190	8694
*R* factor (%)	21.7	22.4
*R*_free_ (%)	27.1	27.9
Average B factor (Å^2^)	55	73
r.m.s. deviation bond lengths^b^ (Å)	0.002	0.004
r.m.s. deviation bond angles^b^ (°)	0.58	0.87
*Molprobity*		
Ramachandran favoured	94%	94%
Ramachandran allowed	5%	5%

^a^Values in brackets show the statistics for the highest resolution shells.

^b^r.m.s. indicates root-mean-square.

## References

[b1] Malumbres M. *et al.* Cyclin-dependent kinases: a family portrait. Nat Cell Biol 11, 1275–6 (2009). 19884882 10.1038/ncb1109-1275 PMC2914104

[b2] Lim S. & Kaldis P. Cdks, cyclins and CKIs: roles beyond cell cycle regulation. Development 140, 3079–93 (2013). 23861057 10.1242/dev.091744

[b3] Mikolcevic P. *et al.* Cyclin-dependent kinase 16/PCTAIRE kinase 1 is activated by cyclin Y and is essential for spermatogenesis. Mol Cell Biol 32, 868–79 (2012). 22184064 10.1128/MCB.06261-11 PMC3272973

[b4] Davidson G. *et al.* Cell cycle control of wnt receptor activation. Dev Cell 17, 788–99 (2009). 20059949 10.1016/j.devcel.2009.11.006

[b5] Mikolcevic P., Rainer J. & Geley S. Orphan kinases turn eccentric: a new class of cyclin Y-activated, membrane-targeted CDKs. Cell Cycle 11, 3758–68 (2012). 22895054 10.4161/cc.21592 PMC3495819

[b6] Evans T., Rosenthal E. T., Youngblom J., Distel D. & Hunt T. Cyclin: a protein specified by maternal mRNA in sea urchin eggs that is destroyed at each cleavage division. Cell 33, 389–96 (1983). 6134587 10.1016/0092-8674(83)90420-8

[b7] Brown N. R., Noble M. E., Endicott J. A. & Johnson L. N. The structural basis for specificity of substrate and recruitment peptides for cyclin-dependent kinases. Nat Cell Biol 1, 438–43 (1999). 10559988 10.1038/15674

[b8] Brown N. R. *et al.* Cyclin B and cyclin A confer different substrate recognition properties on CDK2. Cell Cycle 6, 1350–9 (2007). 17495531 10.4161/cc.6.11.4278

[b9] Schulman B. A., Lindstrom D. L. & Harlow E. Substrate recruitment to cyclin-dependent kinase 2 by a multipurpose docking site on cyclin A. Proc Natl Acad Sci USA 95, 10453–8 (1998). 9724724 10.1073/pnas.95.18.10453 PMC27915

[b10] Fisher R. P. & Morgan D. O. A novel cyclin associates with MO15/CDK7 to form the CDK-activating kinase. Cell 78, 713–24 (1994). 8069918 10.1016/0092-8674(94)90535-5

[b11] Makela T. P. *et al.* A cyclin associated with the CDK-activating kinase MO15. Nature 371, 254–7 (1994). 8078587 10.1038/371254a0

[b12] Ko T. K., Kelly E. & Pines J. CrkRS: a novel conserved Cdc2-related protein kinase that colocalises with SC35 speckles. J Cell Sci 114, 2591–603 (2001). 11683387 10.1242/jcs.114.14.2591

[b13] Marques F. *et al.* A new subfamily of high molecular mass CDC2-related kinases with PITAI/VRE motifs. Biochem Biophys Res Commun 279, 832–7 (2000). 11162436 10.1006/bbrc.2000.4042

[b14] Even Y. *et al.* CDC2L5, a Cdk-like kinase with RS domain, interacts with the ASF/SF2-associated protein p32 and affects splicing *in vivo*. J Cell Biochem 99, 890–904 (2006). 16721827 10.1002/jcb.20986

[b15] Cheng S. W. *et al.* Interaction of cyclin-dependent kinase 12/CrkRS with cyclin K1 is required for the phosphorylation of the C-terminal domain of RNA polymerase II. Mol Cell Biol 32, 4691–704 (2012). 22988298 10.1128/MCB.06267-11 PMC3486194

[b16] Blazek D. *et al.* The Cyclin K/Cdk12 complex maintains genomic stability via regulation of expression of DNA damage response genes. Genes Dev 25, 2158–72 (2011). 22012619 10.1101/gad.16962311 PMC3205586

[b17] Bartkowiak B. *et al.* CDK12 is a transcription elongation-associated CTD kinase, the metazoan ortholog of yeast Ctk1. Genes Dev 24, 2303–16 (2010). 20952539 10.1101/gad.1968210 PMC2956209

[b18] Kohoutek J. & Blazek D. Cyclin K goes with Cdk12 and Cdk13. Cell Div 7, 12 (2012). 22512864 10.1186/1747-1028-7-12 PMC3348076

[b19] Buratowski S. Progression through the RNA polymerase II CTD cycle. Mol Cell 36, 541–6 (2009). 19941815 10.1016/j.molcel.2009.10.019 PMC3232742

[b20] Fuda N. J., Ardehali M. B. & Lis J. T. Defining mechanisms that regulate RNA polymerase II transcription *in vivo*. Nature 461, 186–92 (2009). 19741698 10.1038/nature08449 PMC2833331

[b21] Bowman E. A. & Kelly W. G. RNA polymerase II transcription elongation and Pol II CTD Ser2 phosphorylation: A tail of two kinases. Nucleus 5, 224–36 (2014). 24879308 10.4161/nucl.29347 PMC4133218

[b22] Bosken C. A. *et al.* The structure and substrate specificity of human Cdk12/Cyclin K. Nat Commun 5, 3505 (2014). 24662513 10.1038/ncomms4505 PMC3973122

[b23] Liang K. *et al.* Characterization of human cyclin-dependent kinase 12 (CDK12) and CDK13 complexes in C-terminal domain phosphorylation, gene transcription, and RNA processing. Mol Cell Biol 35, 928–38 (2015). 25561469 10.1128/MCB.01426-14 PMC4333096

[b24] Bartkowiak B. & Greenleaf A. L. Expression, purification, and identification of associated proteins of the full-length hCDK12/CyclinK complex. J Biol Chem 290, 1786–95 (2015). 25429106 10.1074/jbc.M114.612226 PMC4340420

[b25] Davidson L., Muniz L. & West S. 3′ end formation of pre-mRNA and phosphorylation of Ser2 on the RNA polymerase II CTD are reciprocally coupled in human cells. Genes Dev 28, 342–56 (2014). 24478330 10.1101/gad.231274.113 PMC3937513

[b26] Eifler T. T. *et al.* Cyclin-dependent kinase 12 increases 3′ end processing of growth factor-induced c-FOS transcripts. Mol Cell Biol 35, 468–78 (2015). 25384976 10.1128/MCB.01157-14 PMC4272423

[b27] Chen H. H., Wang Y. C. & Fann M. J. Identification and characterization of the CDK12/cyclin L1 complex involved in alternative splicing regulation. Mol Cell Biol 26, 2736–45 (2006). 16537916 10.1128/MCB.26.7.2736-2745.2006 PMC1430317

[b28] Cancer Genome Atlas Research, N. Integrated genomic analyses of ovarian carcinoma. Nature 474, 609–15 (2011). 21720365 10.1038/nature10166 PMC3163504

[b29] Joshi P. M., Sutor S. L., Huntoon C. J. & Karnitz L. M. Ovarian cancer-associated mutations disable catalytic activity of CDK12, a kinase that promotes homologous recombination repair and resistance to cisplatin and poly(ADP-ribose) polymerase inhibitors. J Biol Chem 289, 9247–53 (2014). 24554720 10.1074/jbc.M114.551143 PMC3979363

[b30] Ekumi K. M. *et al.* Ovarian carcinoma CDK12 mutations misregulate expression of DNA repair genes via deficient formation and function of the Cdk12/CycK complex. Nucleic Acids Res 43, 2575–89 (2015). 25712099 10.1093/nar/gkv101 PMC4357706

[b31] Bajrami I. *et al.* Genome-wide profiling of genetic synthetic lethality identifies CDK12 as a novel determinant of PARP1/2 inhibitor sensitivity. Cancer Res 74, 287–97 (2014). 24240700 10.1158/0008-5472.CAN-13-2541 PMC4886090

[b32] Shao H. *et al.* Substituted 4-(thiazol-5-yl)-2-(phenylamino)pyrimidines are highly active CDK9 inhibitors: synthesis, X-ray crystal structures, structure-activity relationship, and anticancer activities. J Med Chem 56, 640–59 (2013). 23301767 10.1021/jm301475f PMC3579313

[b33] Baek K., Brown R. S., Birrane G. & Ladias J. A. Crystal structure of human cyclin K, a positive regulator of cyclin-dependent kinase 9. J Mol Biol 366, 563–73 (2007). 17169370 10.1016/j.jmb.2006.11.057 PMC1852425

[b34] Baumli S. *et al.* The structure of P-TEFb (CDK9/cyclin T1), its complex with flavopiridol and regulation by phosphorylation. EMBO J 27, 1907–18 (2008). 18566585 10.1038/emboj.2008.121 PMC2486423

[b35] Baumli S., Hole A. J., Wang L. Z., Noble M. E. & Endicott J. A. The CDK9 tail determines the reaction pathway of positive transcription elongation factor b. Structure 20, 1788–95 (2012). 22959624 10.1016/j.str.2012.08.011 PMC3469819

[b36] Zhang F., Strand A., Robbins D., Cobb M. H. & Goldsmith E. J. Atomic structure of the MAP kinase ERK2 at 2.3 A resolution. Nature 367, 704–11 (1994). 8107865 10.1038/367704a0

[b37] Tahirov T. H. *et al.* Crystal structure of HIV-1 Tat complexed with human P-TEFb. Nature 465, 747–51 (2010). 20535204 10.1038/nature09131 PMC2885016

[b38] Pinhero R., Liaw P. & Yankulov K. A uniform procedure for the purification of CDK7/CycH/MAT1, CDK8/CycC and CDK9/CycT1. Biol Proced Online 6, 163–172 (2004). 15328539 10.1251/bpo86 PMC514536

[b39] Kabsch W. Xds. Acta Crystallogr D Biol Crystallogr 66, 125–32 (2010). 20124692 10.1107/S0907444909047337 PMC2815665

[b40] Evans P. R. & Murshudov G. N. How good are my data and what is the resolution? Acta Crystallogr D Biol Crystallogr 69, 1204–14 (2013). 23793146 10.1107/S0907444913000061 PMC3689523

[b41] Winn M. D. *et al.* Overview of the CCP4 suite and current developments. Acta Crystallogr D Biol Crystallogr 67, 235–42 (2011). 21460441 10.1107/S0907444910045749 PMC3069738

[b42] McCoy A. J. *et al.* Phaser crystallographic software. J Appl Crystallogr 40, 658–674 (2007). 19461840 10.1107/S0021889807021206 PMC2483472

[b43] Stein N. CHAINSAW: a program for mutating pdb files used as templates in molecular replacement. J. Appl. Cryst. 41, 641–643 (2008).

[b44] Emsley P., Lohkamp B., Scott W. G. & Cowtan K. Features and development of Coot. Acta Crystallogr D Biol Crystallogr 66, 486–501 (2010). 20383002 10.1107/S0907444910007493 PMC2852313

[b45] Murshudov G. N. *et al.* REFMAC5 for the refinement of macromolecular crystal structures. Acta Crystallogr D Biol Crystallogr 67, 355–67 (2011). 21460454 10.1107/S0907444911001314 PMC3069751

[b46] Adams P. D. *et al.* PHENIX: a comprehensive Python-based system for macromolecular structure solution. Acta Crystallogr D Biol Crystallogr 66, 213–21 (2010). 20124702 10.1107/S0907444909052925 PMC2815670

[b47] Chen V. B. *et al.* MolProbity: all-atom structure validation for macromolecular crystallography. Acta Crystallogr D Biol Crystallogr 66, 12–21 (2010). 20057044 10.1107/S0907444909042073 PMC2803126

[b48] Schrödinger LLC. The PyMOL Molecular Graphics System. (Version 1.2r3pre).

